# Pyrozone—It Uses

**Published:** 1897-06

**Authors:** E. A. Honey

**Affiliations:** Kalamazoo


### Pyrozone—Its Uses.

Read before the South-Western Michigan Dental Association.

BY DR. E. A. HONEY, D.D.S., KALAMAZOO.

Pyrozone solutions consist of accurate percentages of $H^2 O^2$ in water and in ether.

The 3 percent or medicinal pyrozone is an aqueous solution containing 3 percent of $H^2 O^2$ representing fifteen volumes of the gas.

The 5 percent or antiseptic pyrozone, is an etherial solution containing 25 volumes of the gas.

The 25 percent solution is what is known as the "Caustic Pyrozone."

As to their uses in dentistry, we will take them up in turn. Not because their uses are distinct from each other, but merely for sake of convenience.

The 3 percent solution is valuable in wiping out root canals of dead teeth; in washing out alveolar abscesses and pus cavities of all kinds; for cleansing the pockets about the necks of teeth after the removal of tartar; also in the treatment of pyorrhea alveolaris. It is sometimes best applied in these cases with the hypodermic syringe, carrying the solution well down in the pockets. It is also used in treating cases of necrosis.

It will be found valuable in removing stain from the teeth, a good method being to moisten the pumice stone with equal parts of pyrozone and water, and apply in the usual manner. This will be found efficient in the vast majority of cases. A few cases will require the full strength, and in individual cases resort will be had to the 5 percent solution. For cleansing the surface of the gums before inserting the hypodermic, the 3 percent solution will be found a convenient agent. It has also been found of value in removing mucus from the surface of the mouth before getting an impression. In cases where the oral fluids are of a thick ropy consistency, this condition disappears on rinsing the mouth with the solution, and the impression can be gotten before the condition returns.

The medicinal pyrozone can also be used to a very limited extent in bleaching devitalized teeth, but is a slow process, as the amount of ogygen liberated is insufficient to do the work. The 5 percent pyrozone is known as the antiseptic solution. It is useful in the cleansing of root canals and pus cavities. The rapid evaporation of the ether makes it a much more powerful agent than the 3 percent solution. Care should be taken in its use, not to allow it to come in contact with the soft tissues. Apply the rubber dam wherever possible. It is used with good results in treating pyrorrhea, but should be used in limited quantities, not allowing it to spread to the healthy tissues. The surrounding mucus membrane can be protected by smearing with glicerine or vaseline, which will relieve the burning and prickling sensation produced by the pyrozone.

The 5 percent solution is also used extensively in bleaching devitalized teeth. In preparing a tooth for bleaching the pulp cavity and the canal up well beyond the neck of the tooth should be carefully cleared of all organic matter ; then with the rubber dam adjusted saturate a pledget of cotton with the solution, place in the cavity, and turn on a blast of warm air, repeating the process until the desired result is obtained. The wiping of the cavity with ammonia previous to applying the pyrozone will dissolve the fatty substances in the cavity and hasten the bleaching process.

The 25 percent pyrozone, or caustic solution, is used principally in pyorrhea, and for bleaching purposes. Bleaching is carried on much more rapidly with the 25 percent solution, as more ogygen is liberated, oxdizing more rapidly the contents of the dental tubuli. In the treatment of pyorrhea with the caustic solution still greater care should be taken to confine its application to the infected parts, as its action on healthy tissues is much more powerful and disagreeable than either of the other solutions.

I will leave the subject at this point, with the hope that in the discussion that is to follow sufficient may be brought out to make the subject of some value to the Society.

DISCUSSION.

Dr. C. R. Rowley: The essayist, Dr. Honey, has given us a short, concise and valuable paper, and in opening the discussion I have penned a few thoughts on paper, that I may read to you, which might otherwise take flight. The study of pyrozone and its dental uses, is a varied and complicated one, principally because the remedy is known to us by numerous names, known to us as dentists for many years as simply P. of H.; but of late years as Pyrozone, Hydrogen Medicinal Ozone, Hydrogen Dioxide, and all chemically known under the symbol of $H_2 O_2$, and other manufacturing pharmacists yet to hear from. I venture the assertion that Dr. Honey would have soured long before he finished his paper, if he had not been so well sweetened, and it is not surprising to me that Dr. Ray is not with us to-day to read us a paper on this subject. I say this in no spirit of levity, as Dr. Ray ever commanded my respect and admiration, but I say the excitement of one, subject to heart trouble, in preparing a paper to be read before this large and intelligent body on a medicinal agent, with synonyms to burn, is a hazardous undertaking. The good Doctor's death undoubtedly deprives us of a very valuable paper, as I know he had made the uses of this agent a special study for many years and could have given us perhaps a better elucidation of pyrozone and its action, than any other member of this society. Begging your pardon for this slight digression, let us discuss the paper just read. In the beginning Dr. Honey tells us nothing from personal knowledge and observation. The personal pronoun "I" is not used once in his paper. We take it for granted, however, that he speaks from facts gained by actual demonstrations. He says pyrozone "3 percent solution is valuable in wiping out root-canals of dead teeth." I do not like the expression, as I do not see how a tooth can be called dead so long as it remains intact, in the mouth, the root covered and partially nourished by the pericementum. But as the expression "dead tooth" is quite generally used for pulpless tooth, we can not, nor do we wish to enter any criticism; but I would not deem it expedient or wise to wash out the canal of a tooth, especially where the pulp is found in a putrescent or suppurating condition, for fear of the boiling properties of the pyrozone bringing particles of septic matter through the foramen, thereby causing irritation, inflammation and alveolar abscess.

The Doctor tells us it is used in treating necrosis, but with what effect he fails to enlighten us. The powerful *effervescing* properties of pyrozone might hasten the exfoliation of the dead from the live bone, by mechanical or chemical pressure, otherwise I do not see wherein its use would be effectual in necrosis. I have found it as the Doctor states, a valuable adjunct in the removal of green stain, especially from children's teeth, and have never observed any injurious after-effects. Would like to hear what the Doctor has to say on that point.

A goodly part of the paper is devoted to bleaching—all wasted energy in my opinion, as I believe in this age of perfected crowns where the dark tooth can be excised and a perfect piece substituted on the root, a tooth that will always hold its color—all time spent in bleaching is but wasted love upon the desert air; but if you must bleach, and if the stain is not caused by some metal post that has been inserted to hold a filling, or by the oxidation of an amalgam filling, pyrozone is the agent that will do the work in the manner so ably described by Dr. Honey. In a series of experiments during the past session of the North-Western University of Chicago, Dental Department, conducted by the Professor of Histology, it was found that micro organisms would live in a 3 or 5 percent solution.

---

FROM ten pounds of corn-meal you can obtain eight pounds of good nourishment; while from the same quantity of potatoes there is less than a pound of nutritive material.

